# Progress towards the Control of Strongyloidiasis in Tropical Australia?

**DOI:** 10.4269/ajtmh.19-0922

**Published:** 2020-01-06

**Authors:** Daniel L. Bourque, Karin Leder

**Affiliations:** 1 Division of Infectious Diseases and Travel Medicine, Mount Auburn Hospital, Cambridge, Massachusetts;; 2 Harvard Medical School, Boston, Massachusetts;; 3 School of Public Health and Preventive Medicine, Monash University, Melbourne, Australia;; 4 Victorian Infectious Diseases Service, Royal Melbourne Hospital at Doherty Institute for Infection and Immunity, Melbourne, Australia

*Strongyloides stercoralis* remains a pathogen of global importance, with up to 100 million people infected with the soil-transmitted helminth.^1^ Strongyloidiasis disproportionately affects vulnerable populations, particularly those who live in poverty and substandard sanitary conditions.^2^ *Strongyloides stercoralis* is most commonly transmitted through contact with contaminated soil, which initiates its complex human life cycle, culminating in filariform larvae reaching the small intestine, where they mature to adult worms. This sets up the autoinfection life cycle of *S. stercoralis*, which allows it to persist in a human host for decades. In the setting of compromised cell-mediated immunity, hyperinfection may occur, with widespread dissemination, which carries a high mortality rate.^3^ Infection with *S. stercoralis* and other soil-transmitted helminths has been associated with malnutrition, growth stunting, and cognitive impairment in children.^4^ Although improving sanitation and reducing environmental fecal contamination remains an essential component to the control of strongyloidiasis and other parasitic infections, the high global burden of disease has galvanized alternative approaches, ranging from mass drug administration (MDA) to targeted test and treat approaches. However, these control programs are hampered by the lack of robust prevalence data in regions where *S. stercoralis* is endemic.

In this issue of the journal, Paltridge and others^5^ describe demographic and temporospatial changes seen in opportunistic serological testing for strongyloidiasis in the Far North Queensland (FNQ) region of Australia over a 19-year period (2000–2018). In this study of nearly 2,500 individuals, the proportion of positive serology tests fell significantly, with a concomitant increase in ivermectin prescribing. They also describe the comparative seropositivity among indigenous versus nonindigenous Australians, with the former having almost four times the odds of seropositivity.

The authors briefly acknowledge the two main limitations of the study. First, it is a retrospective study, with a consequent possibility of selection bias. Indeed, although no population rates are provided, the number of tests included in the report doubled from an average of 97 per year in the first 5 years (2000–2004) to an average of 190 annually in the last 5 years (2014–2018). This means that the findings could reflect changes in testing practices and greater recognition of the importance of screening for infection among “lower risk” individuals (e.g., before initiation of immunosuppression) leading to an apparent drop in the proportion of positive tests rather than a true change in infection prevalence. Similarly, the reported apparent “closing of the gap” between indigenous and nonindigenous Australians, based on the proportions of positive tests in each group, could represent unequal access to testing services, with an average of 84% of those tested in 2000–2004 being indigenous, versus 52% in 2014–2018. This could mean that the most vulnerable are increasingly being missed from screening and would infer a more pessimistic conclusion about the health of indigenous individuals than that reached by the authors.

A second limitation is the use of serology alone for diagnosis of strongyloidiasis. Imperfect sensitivity and specificity of the commonly used serological tests, especially in the context of potential cross-reactivity from coinfection with other helminths, impedes interpretability of infection status and treatment response on an individual level. However, microscopy and culture techniques are relatively labor intensive and insensitive (particularly on single fecal specimens), and polymerase chain reaction availability remains limited. Therefore, for population-level seroprevalence measures and trends over time, increased serological testing could provide better population prevalence data and facilitate targeted interventions. Improved data on *Strongyloides*-associated morbidity among Australian indigenous populations might also help alleviate the controversy that exists among health professionals regarding likely benefits of MDA programs and concerted efforts at environmental control.^6^

Despite these limitations, this study suggests that there may be a decline in the prevalence of strongyloidiasis in the FNQ region of Australia. The explanation for this is unclear, although changes may reflect improvements in sanitation and other environmental control measures in Northern Australia. However, considering that humans are likely the predominant reservoir, the increased use of ivermectin may also have been a significant driver of any decrease in strongyloidiasis prevalence. Another study in tropical Australia demonstrated that after treatment with ivermectin there was a sustained decrease in the rates of *S. stercoralis* seropositivity at 3 years of follow-up.^7^ Other studies have found similar results.^8,9^ This suggests that addressing the human reservoir may lead to lasting benefits for the general population and indicates that with effective programs aimed at *S. stercoralis* infection, elimination may be an attainable goal. Moreover, a single dose of ivermectin was recently shown to be as effective as a multidose regimen for the treatment of strongyloidiasis, supporting a simplified approach to test and treat approaches or for MDA programs.^10^ Considering this, the findings outlined by Paltridge et al. likely reflect the combination of scaling up of ivermectin prescribing coupled with improvements in sanitation.

Recognizing the robust potential of test and treat approaches using a single dose of ivermectin, there is a need for more precise data on the prevalence of strongyloidiasis in endemic regions such as Northern Australia. This is especially important in areas where human T-lymphotropic virus type I coinfection is common. Identifying areas of high prevalence will allow for effective deployment of interventions to work toward reducing the morbidity of infection, and ultimately toward the improved control of strongyloidiasis in the general population. However, this will likely require a concerted effort involving high-risk communities, health-care practitioners, and public health officials, along with rigorous diagnostic assessment through the use of serology, stool diagnostic methods, and also more sensitive molecular-based testing in a non-biased fashion. Furthermore, reducing the worm burden, although an acceptable goal for other helminths, is insufficient for strongyloidiasis because of the auto-infection cycle, and so posttreatment monitoring may be needed. Without these efforts, it is unlikely that an effective control program will be achieved. Although there may be progress in tropical Australia and other regions, elimination of strongyloidiasis remains a distant goal.
